# Supraorbital Blowin Fracture Presenting as an Ocular Dystopia in a Nine-Year-Old Girl

**DOI:** 10.1155/2013/574146

**Published:** 2013-07-15

**Authors:** Ranganadh Nallamothu, Shanmukha Reddy Kallam, Srikanth Gunturu, Sukumar Singh, Vijay Kumar Rachalapally

**Affiliations:** ^1^Department of Oral and Maxillofacial Surgery, Drs. Sudha & Nageswarao Siddhartha Institute of Dental Sciences, Chinoutpalli, Gannavaram 521 286, Krishna District, Andhra Pradesh, India; ^2^Sri Sai College of Dental Surgery, Shiv Nagar, Kotherapally, Range Reddy District, Vikarabad 501101, Andhra Pradesh, India; ^3^A B Shetty Memorial Institute of Dental Sciences Medical Sciences Complex, P.O. Nityanandanagar, Deralakatte 575018, India; ^4^Subharati Dental College, Meerut Subharati Puram, NH-58, Delhi-Haridwar Bypass Road, Meerut 250005, Uttar Pradesh, India; ^5^S.V.S. Institute of Dental Sciences, Mahabubnagar 509 001, Andhra Pradesh, India

## Abstract

A 9-year-old girl was referred to a trauma centre with severe head injury. 3D CT scan revealed depressed fracture involving the frontal bone on the right side, right parietal bone, and right superior orbital margin, right lamina papyracea. The frontal table was managed conservatively and open reduction and internal fixation was done for the supraorbital blow in to correct the ocular dystopia. The clinical course, possible mechanism, and management of the patient are discussed.

## 1. Introduction

Head injury is a common sequelae in the road traffic accident. Fractures of the supraorbital region are rare and are frequently associated with high-energy craniomaxillofacial trauma. When displacement of the orbital roof occurs, exploration and precise reconstruction are warranted to limit such ocular complications as exophthalmos, enophthalmos, proptosis, dystopia (ocular and orbital), diplopia, restricted ocular movement, altered vision, pain, and discomfort [1–13]. Fractures of the supraorbital rim can result in significant ophthalmologic and cosmetic morbidity. Isolated supraorbital rim fractures are rare [14, 15]. However, an estimated 1% to 9% of facial fractures can involve the supraorbital rims and the anterior table of the frontal sinus, and many supraorbital rim fractures are associated with other forms of craniomaxillofacial injury [1–6, 13]. Many of these patients have multisystem injuries, most of which are neurologic [16, 17]. These fractures are associated with high-energy impacts, motor vehicle collisions being the most frequently reported etiology [1, 18]. Many other causes have been identified, including tire explosions, ruptured garage door springs, chain saws, high-voltage electric shocks, swinging objects, and falls from high places [1, 3, 9, 10, 18, 19].

Patients with supraorbital rim fractures have characteristic physical signs and symptoms [1, 2, 12, 18]. If they are seen soon after the traumatic episode, then a cosmetic deformity consisting of depression or flattening of the supraorbital ridge can be visualized. Later, these injuries may present with intensely turgid periorbital ecchymosis, edema, soft tissue lacerations, and paresthesia over the area of distribution of the supraorbital and supratrochlear nerves. If the fracture is displaced, dystopia (ocular and orbital), enophthalmos, exophthalmos, and proptosis may be noted, along with diplopia [12]. Ocular discomfort, epiphora, limitation of eye movement, increased scleral show, and increased width of the palpebral fissure have all been reported [12].

A review of the literature reveals no uniform system for the classification of supraorbital rim fractures; most authors rely on descriptive terminology. A nondisplaced supraorbital rim fracture generally requires no surgical intervention [20, 21]. An orbital roof fracture, with undisplaced supraorbital rim involvement and no frontal sinus fracture, is common in children [22]. When the fractured segments are displaced, surgical exploration, reduction, and stabilization are indicated. Supraorbital rim fractures frequently involve the frontal sinus. If the anterior table of the frontal sinus and the supraorbital rim was displaced, then operative treatment is required [14, 15].

A computed tomography (CT) scan can rule out damage to the posterior table of the frontal sinus. If there is a displaced fracture of the posterior table, then a dural tear is quite possible. However, treatment of such an injury is beyond the scope of this paper and must be carried out by a neurosurgeon of the team. The need for fixation in supraorbital rim fractures depends on the type of fracture encountered. The reduction is often stable once the fragments have been levered into position because of the absence of muscular displacing forces [14]. The introduction of rigid fixation into craniomaxillofacial fracture management revolutionized the treatment of orbital injuries [23].

## 2. Case Report

**Figure 1:**
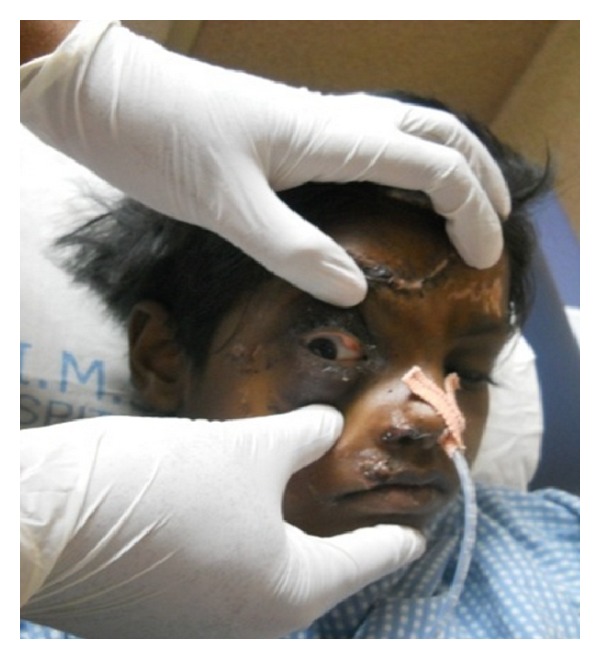
Subconjunctival hemorrhage, circumorbital ecchymosis, and horizontal ocular dystopia involving the right eye.

A nine-year-old girl reported to a trauma centre with head injury with history of loss consciousness at the time of trauma and brought semiconscious to the hospital GCS-E1M5V2. On examination, she had a laceration on the frontal aspect of the face, right supraorbital region, and some abrasions on the chest. Subconjunctival hemorrhage, circumorbital ecchymosis, and horizontal ocular dystopia involving the right eye were observed (Figure 1). Right-eye ball was displaced to the lateral side of the orbit. 3D CT scan revealed depressed fracture involving the frontal bone on the right side, right parietal bone, right superior orbital margin, right lamina papyracea, right maxillary, bilateral ethmoid, sphenoid sinusitis/hemosinus, and small posttraumatic encephalocele at anterior skull base from cribriform plate. 

**Figure 2:**
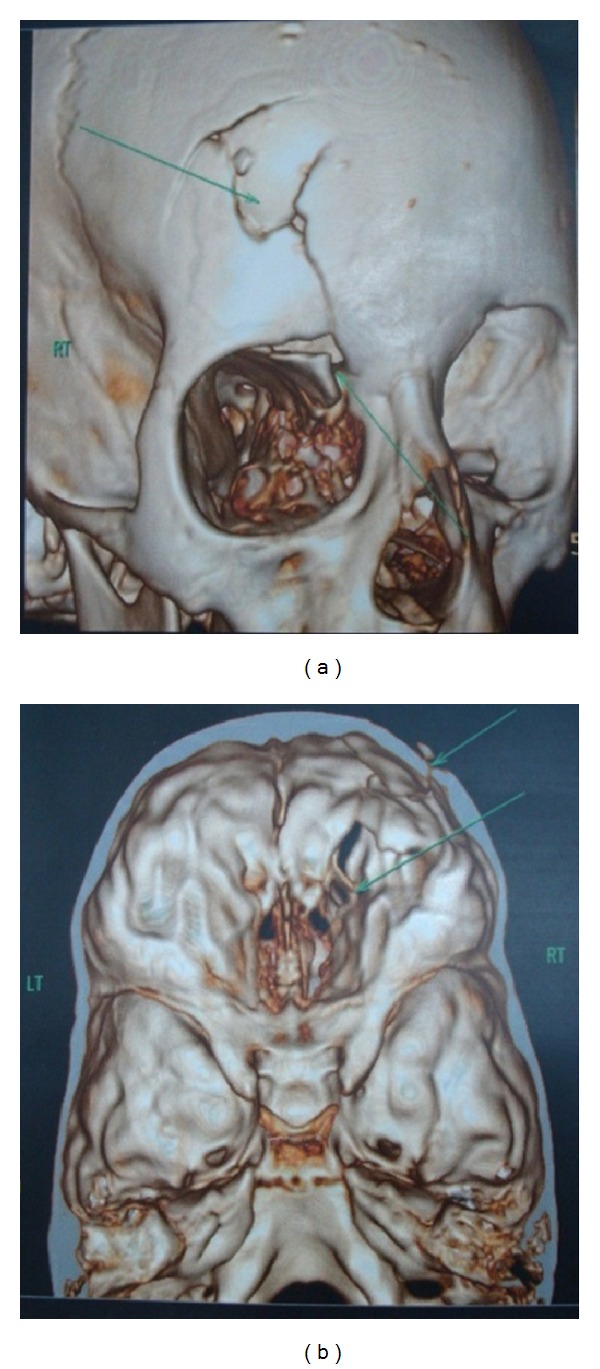
3D CT scan revealed depressed fracture involving the frontal bone on the right side, right parietal bone, right superior orbital margin, right lamina papyracea, right maxillary, bilateral ethmoid, sphenoid sinusitis/hemosinus, and small posttraumatic encephalocele at anterior skull base from cribriform plate.

**Figure 3:**
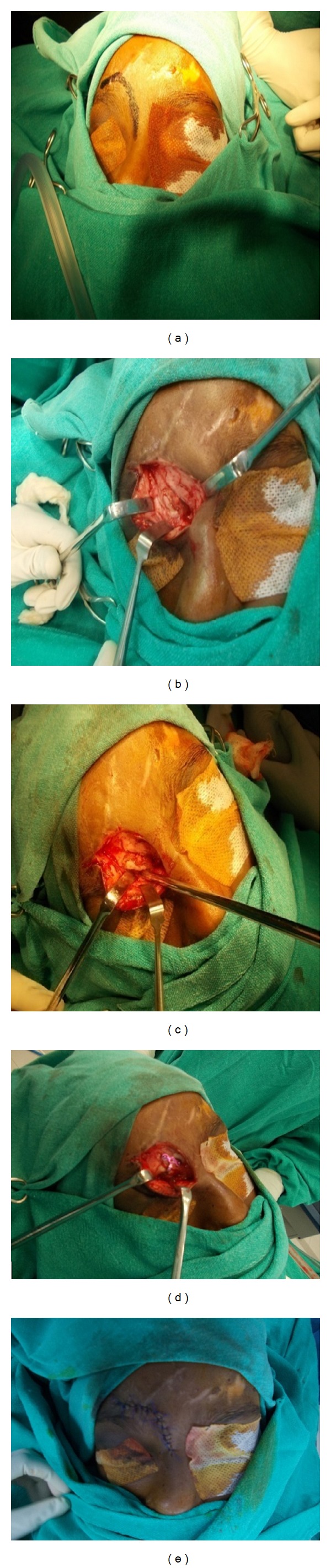
(a) Marking for the incision. (b) Fracture site exposed. (c) Reducing the segment manually with Howarth's periosteal elevator. (d) Open reduction and internal fixation with titanium plate and screws. (e) Layerwise closure done.

Soft tissue swelling involving right orbitonasal and frontal region (Figures 2(a) and 2(b)). Frontal laceration was sutured primarily by a plastic surgeon at the primary stage. Later the patient was operated for the correction of the ocular dystopia. It was noticed that the supraorbital rim (medial 1/3) was pushing the globe from medial to lateral direction, and there was a step deformity on the right frontal aspect. Open reduction and internal fixation of supraorbital margin were planned under GA with nasal intubation. Supraorbital rim was approached through the existing scar with extension to the glabella region (Figures 3(a)–3(c)).

**Figure 4:**
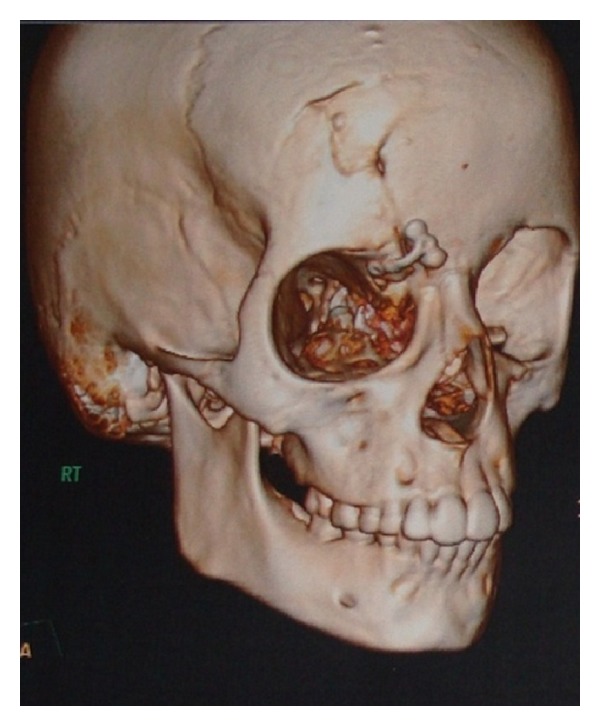
Postoperative 3D CT scan revealing titanium plate and screws intact at the fracture site.

**Figure 5:**
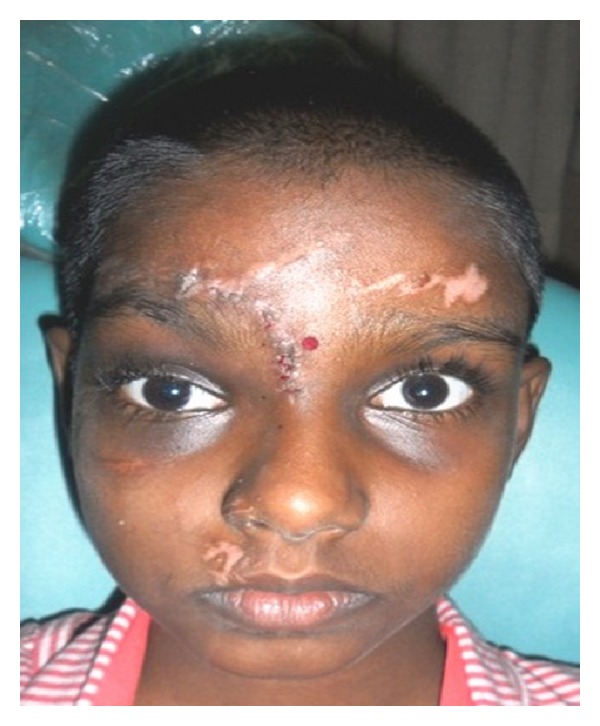
Two weeks after operation.

Dissection was carried out layerwise, the displaced supraorbital bony part was identified. It was noticed that a part of brain tissue was herniating between the two fracture segments; so an attempt was made to push the brain tissue, and the two fracture segments were reduced manually. A T-shaped titanium plate was bent according to contour of supraorbital roof and supraorbital ridge; horizontal bar of the plate was fixed on the supraorbital margin with 1.5 mm screws, and vertical bar was fixed to the roof with one 1.5 mm screw. Layerwise closure was done (3–0 vicryl for submucosal and 5–0 prolene for skin), and the recovery was uneventful. Frontal bone fracture was managed conservatively. Postoperative 3D CT scan was advised to confirm the reduction (Figure 4). Patient was discharged after 5 days and reviewed every 4 weeks. After one month postoperatively the patient was reviewed (Figure 5). Upon examination, it was found that the healing and the ocular dystopia were corrected satisfactorily.

## 3. Discussion

### 3.1. Orbital Roof Fractures: Pathophysiology

There are several different configurations of orbital roof fractures including nondisplaced, isolated “blowin,” isolated “blowout” (or “blowup”), supraorbital rim involvement (without frontal sinus), frontal sinus involvement, and combination fracture [24]. The common mechanism of injury for a superior orbital fracture is high-energy, blunt trauma to the orbit or forehead. The fracture is generally the result of direct extension of a force vector into the site of fracture, or due to a transient increase in orbital or intracranial pressure that results in fracture of the orbital roof. 

The isolated orbital roof “blowup” fracture, also known as “blowout” fracture, is defined as superior displacement of the fracture fragment into the anterior cranial fossa without involvement of the supraorbital rim, with possible herniation of orbital contents outside of the orbital confines [24]. The isolated “blowup” fracture is thought to be the result of direct orbital blunt force with subsequent increased intraorbital pressure, hydraulic forces, and/or shear strain [24]. 

The isolated “blowin” fracture is defined as inferior displacement of the roof without involvement of the supraorbital rim or the frontal sinus and is thought to be the result of increased intracranial pressure, a shift of the cranium, and/or a shift of the intracranial contents [24]. The blowin fracture effectively reduces the volume of the orbit and can cause associated intraorbital injuries including extraocular muscle entrapment and optic nerve injury. Although the terms “blowin” and “blowup” fractures refer to isolated injuries of the internal superior orbit, these injuries occur far more commonly in conjunction with supraorbital rim and frontal sinus involvement [24]. 

When other craniofacial injuries are identified, it is thought that the mechanism of injury is direct transmission of force from displacement of the adjacent injury [24]. Very rarely, the orbital roof will fracture without displacement of fractures fragments, resulting in the nondisplaced orbital roof fracture [24]. 

## 4. Treatment

Orbital roof fractures are typically managed by otolaryngologists, ophthalmologists, neurosurgeons, plastic surgeons, and/or oral-maxillofacial surgeons depending on the individual case and associated imaging/clinical findings. Generally speaking, pediatric orbital roof fractures are less likely to require surgical repair than their adult counterpart [24, 25]. Currently, there is no specific consensus on the treatment of orbital fractures in the pediatric population [26].

Surgery is often performed if significant neurological, ophthalmologic, or aesthetic deficiency is clinically apparent or expected to eventually result from the injury, and the surgical intervention is likely to improve clinical outcomes. In general, surgical intervention is utilized only to repair displaced and comminuted fractures that will likely cause functional disability, cosmetic deformity, or both [27]. Pure “blowin” fractures, “blowout” fractures, and nondisplaced fractures that are asymptomatic generally have minimal clinical consequences and can be managed conservatively without surgery [26]. Fractures that extend beyond the orbital roof can generally be treated conservatively with diligent clinical and CT followup [26]. 

Surgical intervention is not an entirely benign solution with postoperative complications including enophthalmos, ocular dystopia, extraocular muscle entrapment, infection, orbital volume discrepancy, and blindness [24, 26].
